# Surgical management of syringomyelia associated with spinal arachnoid web: strategies and outcomes

**DOI:** 10.1007/s10143-023-02071-8

**Published:** 2023-06-26

**Authors:** Sasan Darius Adib, Jens Schittenhelm, Peter Kurucz, Till-Karsten Hauser, Marcos Tatagiba

**Affiliations:** 1 Department of Neurosurgery, University of Tuebingen, Hoppe-Seyler-Straße 3, 72076 Tuebingen, Germany; 2 Department of Neuropathology, University of Tuebingen, Hoppe-Seyler-Straße 3, 72076 Tuebingen, Germany; 3 Department of Neurosurgery, Klinikum Stuttgart, Kriegsbergstraße 60, 70174 Stuttgart, Germany; 4 Department of Neuroradiology, University of Tuebingen, Hoppe-Seyler-Straße 3, 72076 Tuebingen, Germany

**Keywords:** Spinal arachnoid web, Syringomyelia, Scalpel sign, CSF flow, Venturi effect, Arachnopathy

## Abstract

Spinal arachnoid web (SAW) is a rare disease entity characterized as band-like arachnoid tissue that can cause spinal cord compression and syringomyelia. This study aimed to analyze the surgical management of the spinal arachnoid web in patients with syringomyelia, focusing on surgical strategies and outcomes. A total of 135 patients with syringomyelia underwent surgery at our department between November 2003 and December 2022. All patients underwent magnetic resonance imaging (MRI), with a special syringomyelia protocol (including TrueFISP and CINE), and electrophysiology. Among these patients, we searched for patients with SAW with syringomyelia following careful analysis of neuroradiological data and surgical reports. The criteria for SAW were as follows: displacement of the spinal cord, disturbed but preserved CSF flow, and intraoperative arachnoid web. Patients were evaluated for initial symptoms, surgical strategies, and complications by reviewing surgical reports, patient documents, neuroradiological data, and follow-up data. Of the 135 patients, 3 (2.22%) fulfilled the SAW criteria. The mean patient age was 51.67 ± 8.33 years. Two patients were male, and one was female. The affected levels were T2/3, T6, and T8. Excision of the arachnoid web was performed in all cases. No significant change in intraoperative monitoring was noted. Postoperatively, none of the patients presented new neurological symptoms. The MRI 3 months after surgery revealed that the syringomyelia improved in all cases, and caliber variation of the spinal cord could not be detected anymore. All clinical symptoms improved. In summary, SAW can be safely treated by surgery. Even though syringomyelia usually improves on MRI and symptoms also improve, residual symptoms might be observed. We advocate for clear criteria for the diagnosis of SAW and a standardized diagnostic (MRI including TrueFISP and CINE).

## Introduction

Spinal arachnoid web (SAW) is a rare disease [3, 23] characterized by band-like arachnoid tissue [2] that most often develops at the upper thoracic level and can cause spinal cord compression with myelopathy and syringomyelia. SAW is diagnosed by a displacement of the spinal cord, with intact ventral dura mater and disturbed but conserved cerebrospinal fluid (CSF) flow [23]. It can be differentiated from other disease entities with the ventral displacement of the spinal cord, such as spinal arachnoid cysts (SAC), idiopathic spinal cord herniation (ISCH), and cord adhesion [7].

Mallucci et al. [17] were the first to describe the condition in a series of SAW in 1997 [17, 23], and as at July 2021, only 63 reports have been described in the literature [2].

SAW is believed to originate from the septum posticum [7, 17, 23], ,and it had been also described as an abnormal thickening [11] and/or abnormal formation [1, 3] of the arachnoid membrane. Other authors [19] characterized it as intradural, extramedullary transverse bands that extend from the pial surface of the dorsal aspect of the spinal cord [1, 3], which affect focal pressure on the spinal cord, resulting in dorsal indentation [1, 11, 22] and disturbed CSF flow.

Chellathurai et al. [7] reviewed magnetic resonance imaging (MRI) images of the dorsal spine of 1350 patients and found 28 cases of ventral displacement of the dorsal spinal cord, including 6 cases of SAW. In their study, Nisson et al. [21] concluded that 95% of SAW cases were located dorsally, with 4% located ventrally and 4% located circumferentially. Voglis et al. [31] and Nisson et al. [21] reported that 100% of SAWs were in the thoracic spine, with 67% and 72% of the patients, respectively, being male. In contrast, Hamilton et al. [11] reported a 2:1 female:male predominance. Aiyer et al. [1] noted that patients’ ages ranged from the 4th to 7th decade, while Nisson et al. [21] reported a mean age of 52 years, and Voglis et al. [31] reported a mean age of 54 ± 12.7 years.

This study aimed to analyze the surgical management of SAW in patients with syringomyelia, focusing on surgical strategy and outcome.

## Materials and methods

### Data collection and inclusion criteria

A total of 135 patients with syringomyelia (not included were patients with neoplastic lesions associated with syringomyelia) underwent surgery at the department of Neurosurgery of the University of Tübingen between November 2003 and December 2022 (Table 1). Three patients had syringomyelia due to SAW and are the subject of the present study. They were identified via careful retrospective analysis of neuroradiological data and surgical reports. The criteria for SAW were as follows: displacement of the spinal cord, disturbed but preserved CSF flow, and intraoperative arachnoid web. We excluded patients with spinal cord herniation, spinal arachnoid cysts, cord adhesion, and intramedullary tumors.

**Table 1:** Etiology of syringomyelia (non-neoplastic)

Etiology of syrinx (non-neoplastic)	Number of patients	Percentage (%)
Chiari malformation	65	48.14
Posttraumatic	20	14.81
Arachnoid cyst	17	12.59
Arachnopathy and/or adherence of spinal cord of unknown origin	8	5.93
Post-infectious/associated with meningitis	7	5.19
Post-surgery	4	2.96
Tethered cord	4	2.96
Spinal arachnoid web	3	2.22
Idiopathic syringomyelia	3	2.22
Arachnopathy after bleeding of idopathic origin	2	1.48
Spinal cord herniation	1	0.74
Scoliosis	1	0.74
Total	**135**	**100**

Patients were evaluated for initial symptoms, surgical strategies, and complications by reviewing surgical reports, patient documents, neuroradiological data, and follow-up data.

The study was approved by the ethics committee.

### Preoperative MRI image analysis

All patients underwent MRI with a special syringomyelia protocol (including TrueFISP and CINE). This special syringomyelia protocol is part of our standard operating procedure (SOP) for all patients with syringomyelia, which are treated in our department.

**Fig. 1:**
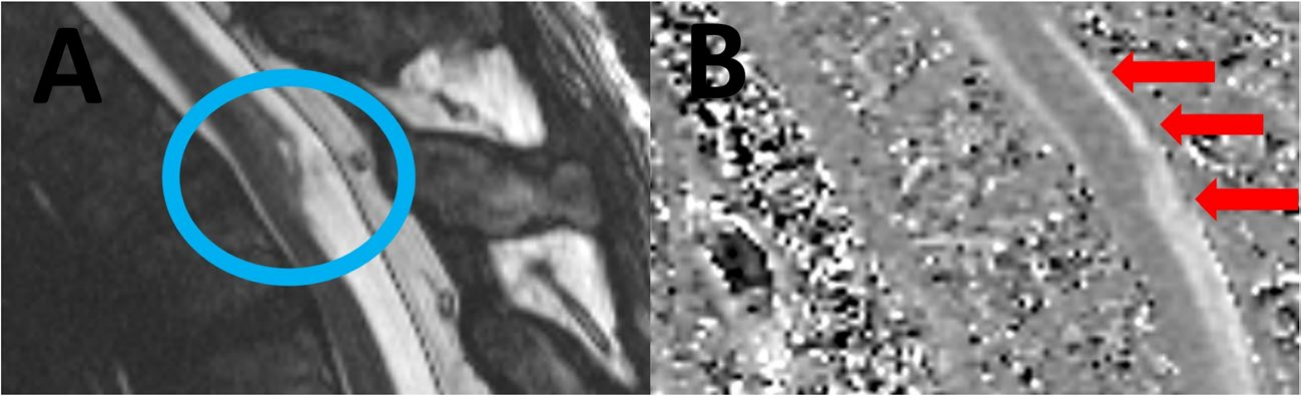
Preoperative sagittal T2-weighted magnetic resonance images (**A**) and CINE (**B**) revealed displacement and caliber variation of spinal cord (scalpel sign) (blue circle), and remnant CSF flow (red block arrows), without any signs of a archnoid cyst.

MRI images were analyzed regarding the displacement of the spinal cord (Fig. 1A), enlargement of the posterior subarachnoid space, and side and level of SAW.

MRI CINE was analyzed regarding remnant CSF flow (Fig. 1B). The axial TrueFISP sequence was analyzed for the detection of SAW.

### Level, size, and volume of syringomyelia

The syringomyelia was analyzed regarding level and extension, length, maximal AP diameter, and maximal lateral diameter in the MRI TrueFISP sequence before and after surgery.

The level of the syrinx, extension in length, and maximal AP and lateral diameters were measured in the MRI TrueFISP sequence before and after surgery. Furthermore, volumetry of the syrinx before and after surgery was performed. For volumetric analyses, axial MRI TrueFISP scans were transmitted to iplan 7 (brainlab®). The syringomyelia was contoured manually on each slice in the TrueFISP images.

### Electrophysiology

Electrophysiological data before and during surgery were analyzed (Motor evoked potentials (MEP), somatosensory evoked potentials (SEP), and silent periods).

### Surgical steps/surgical strategy

Surgical steps were analyzed by reviewing the approach used, the approach level, extension of the approach, intraoperative intradural findings, whether an arachnoid membrane covered the spinal cord, and management of SAW.

### Histopathological evaluation

Surgically resected sample in one case was formalin-fixated, paraffin-embedded and histolologically reviewed on HE stains. Immunohistochemical staining for CD3 was done with a Ventana BenchMark immunostainer (Ventana Medical Systems, Tucson, Arizona, USA).

### Follow-up

Symptoms and clinical examination for motor and sensory function were analyzed in all cases 3 months after surgery and during the last follow-up after surgery.

## Results

Of the 135 patients, who received surgery due to syringomyelia (not included were patients with neoplastic lesions associated with syringomyelia), 3 (2.22%) fulfilled the criteria for SAW. The mean patient age was 51.67 ± 8.33 years. Two patients were male, and one was female (Table 2).

**Table 2:** Initial symptoms, surgical management, complications, follow-up, and recurrence

Case number	Age	Sex	Initial symptoms	Level of SAW	Approach	Surgical strategy	IOM	Complications	FU (months)	Recurrence
1	61	M	Pain in both arms and ventral thorax, palsy left arm	T 6	Laminoplasty (two levels)	Excision of SAW	No change	None	6	No
2	49	F	Palsy of left leg, paresthesia in both feet, slight ataxia	T 2/3	Laminoplasty (one level)	Excision of SAW	No change	None	17	No
3	45	M	Dysesthesia and paresthesia of the right arm	T 8	Laminectomy (one level)	Excision of SAW	No change	None	77	No

### Presenting symptoms

- Case 1: This 61-year-old man presented with pain in both arms and the ventral thorax and a slight palsy of the left arm. The patient had a history of transient ischemic attack, arterial hypertension, status post-Lyme disease, and cervical disk herniation (conservative management).
- Case 2: This 49-year-old woman presented at our outpatient clinic with palsy of the left leg (grade 4/5), paresthesia in both feet, and slight spinal ataxia.
- Case 3: This 45-year-old man presented at our outpatient clinic with dysesthesia and paresthesia of the right arm. The patient had a history of ventral discectomy at the C5/C6 level with cage implantation.

### Radiological findings

**Fig. 2:**
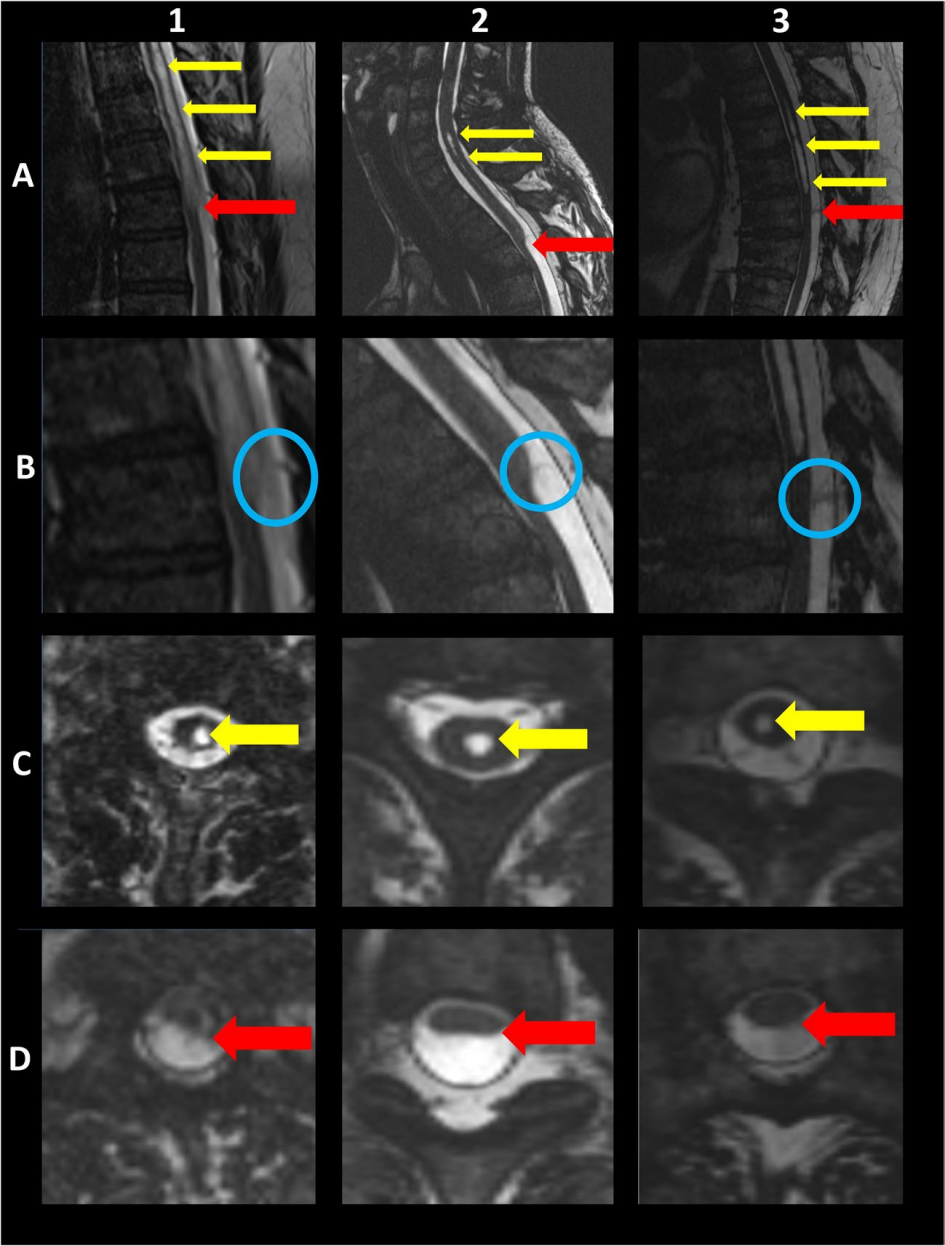
Preoperative magnetic resonance images of patient 1–3. **A** Sagittal MRI revealed displacement and caliber variation of spinal cord (red block arrows) and syringymyelia rostral to the SAW (yellow block arrows). **B** Sagittal MRI TrueFISP revealed a web in all three cases (blue circle). **C** Axial MRI TrueFISP of the syrinx (yellow block arrows). **D** Axial MRI TrueFISP of discplacemnt and caliber variation of spinal cord (red block arrows)

All patients underwent pre- and post-operative MRI (syrinx protocol). Anterior spinal cord displacement was seen in all cases before surgery (Fig. 2A), with enlargement of the posterior subarachnoid spaces. The affected levels were T6 (case 1), T2/3 (case 2), and T8 (case 3) (Table 2). MRI revealed no arachnoid cysts, mass-like lesions, dural defects, or spinal cord herniation in all cases.

A reduced spinal cord caliber at the SAW level was noted in all cases (Fig. 2A, B, D), with the characteristic scalpel sign (in case 3, only a slight scalpel sign was noted). The caliber variation in the level of the SAW was 3 mm, 5 mm, and 1 mm (mean: 3 mm).

Phase-contrast CINE MRI revealed preserved but disturbed CSF flow in all cases. The T2-TrueFISP sequence in sagittal MRI revealed a web in all three cases (Fig. 2B) at the level of caliber change.

### Electrophysiological examination before surgery

- Patient 1: MEPs of the abductor digiti minimi and tibialis anterior muscles and SEPs of the median and tibial nerves revealed the central sensory pathways of the right lower extremity were slightly affected. Cortical silent periods (COSP) revealed inhibition, but cutaneous silent periods (CSP) were not detected on the left side.
- Patient 2: MEPs of the right leg were impaired. SEPs, COSP, and CSP were normal.
- Patient 3: COSP and CSP were normal. The mixed nerve silent period (MNSP) was not detectable on the left side.

### Surgical strategy

All patients were positioned under general anesthesia in the prone position. Intraoperative monitoring included MEP, SEP of the upper and lower extremities, and silent periods.

Two patients underwent laminoplasty (Table 2): one patient at two levels (case 1) and the other at one level, with undercutting of both adjacent laminae (case 2). One patient underwent laminectomy at one level (case 3) (Table 2).

**Fig. 3:**
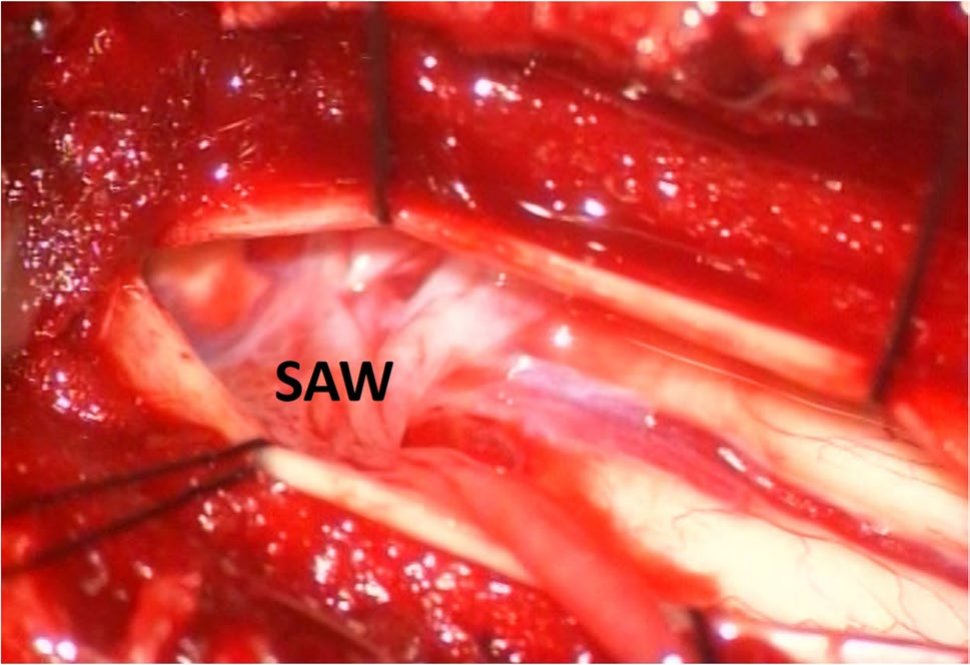
Identification of SAW and caliber variation of spinal cord during surgery

After opening the dura and identifying SAW, we dissected the arachnoid membrane and adhesion bands covering the spinal cord (Fig. 3). The spinal cord compression was resolved after dissection, and CSF flow could be observed.

No significant change in intraoperative monitoring was noted in all cases during surgery.

In one patient (case 1), the membrane underwent histopathological examination.

### Complications

No complications were observed in our series (Table 2).

### Postoperative course, 3 months follow-up, and long-term follow-up

**Fig. 4:**
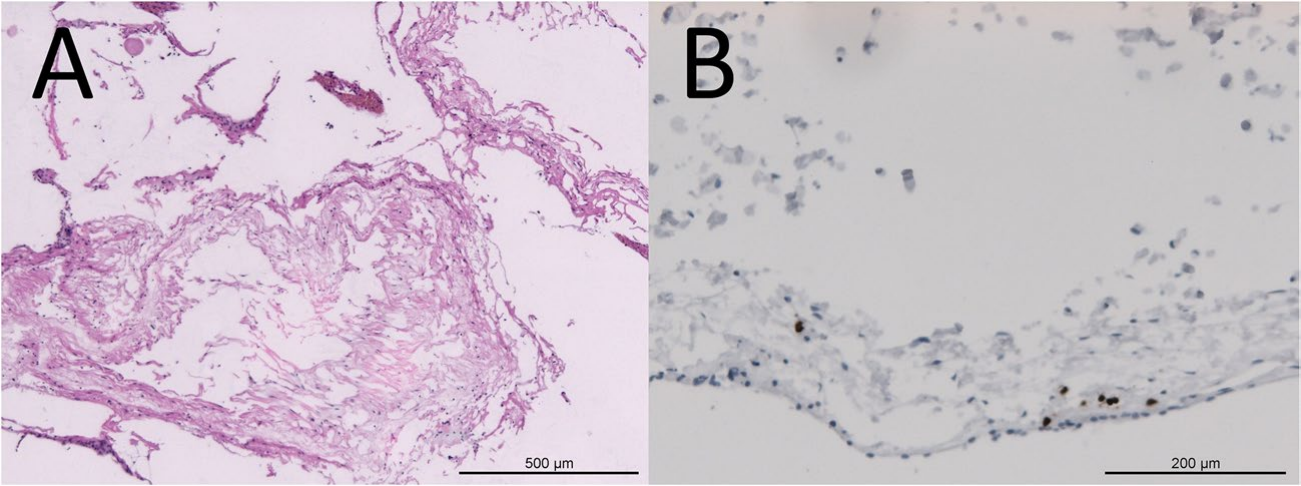
Histopathological findings of SAW revealed fibrotic thickened leptomeningeal tissue with arachnoid cap cell layer (HE, ×40 magnification) (**A**) and small numbers of CD3+ T cells (diaminobenzidine as brown chromogen, CD3 immunohistochemistry, ×100 magnification) (**B**)

Histopathological analysis of SAW in case 1 revealed fibrotic, thickened leptomeningeal tissue layered with arachnoid cap cells (Fig. 4A) and small numbers of infiltrating CD3+ T cells (Fig. 4B). Postoperatively, none of the patients had new neurological symptoms. Most symptoms improved after 3 months. Patients 1 and 3 showed improvement in all symptoms after 3 months. Patient 2 showed only partially improved symptoms, with remnant symptoms after 3 months.

**Fig. 5:**
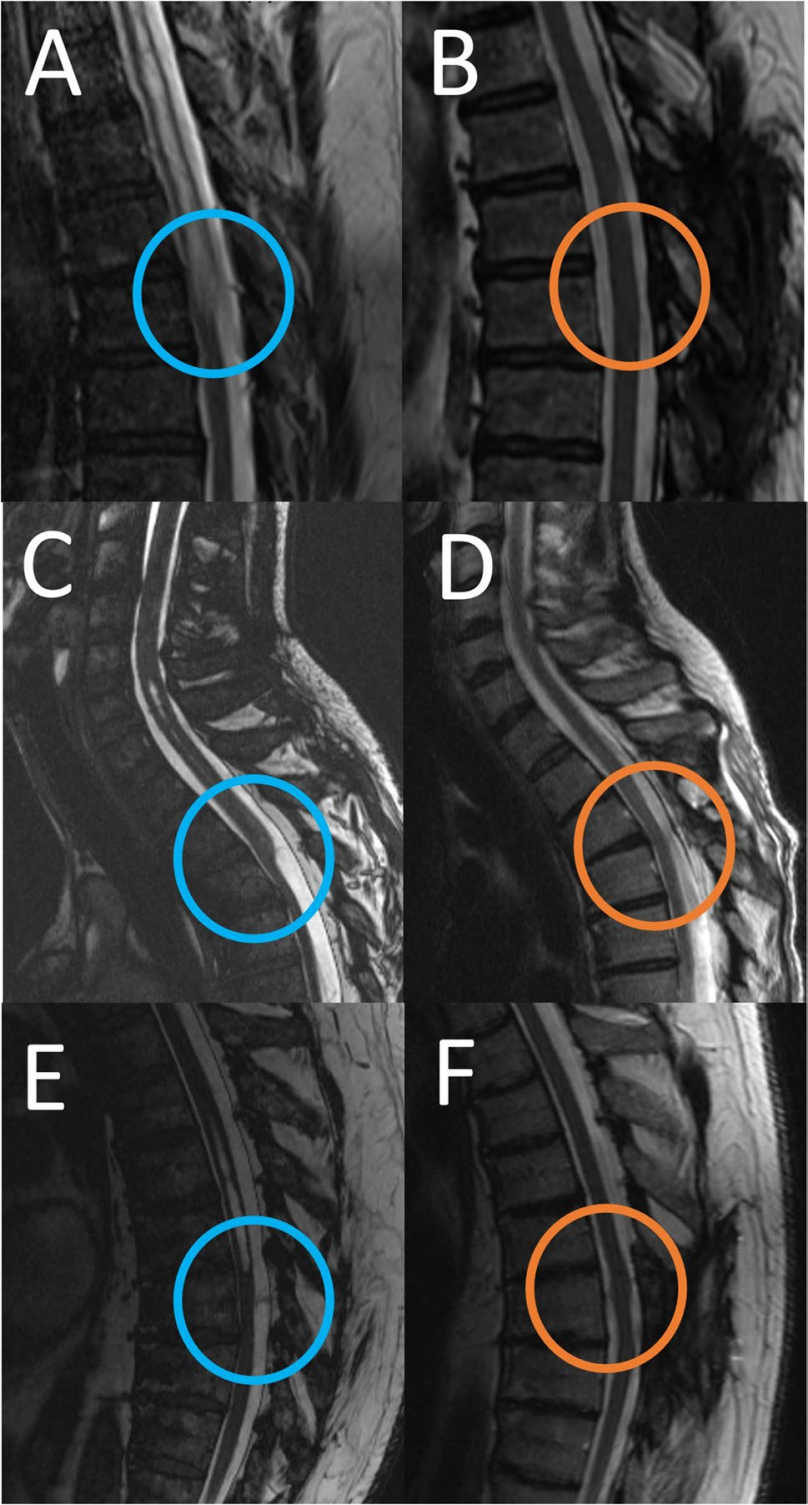
Preoperative (**A**, **C** ,**E**) and postoperative (**B**, **D**, **E**) sagittal MRI of the three patients with SAW. The preoperative caliber variation (blue circle) improved 3 months after surgery (orange circle)

In all three cases, an MRI 3 months after surgery revealed no caliber variation of the spinal cord. The scalpel sign had disappeared (Fig. 5). In two cases (case 1 and case 3), no syringomyelia was detectable. In case two, the syringomyelia improved.

The same clinical and radiological findings were observed in long-term follow-up. The mean long-term follow-up duration was 33.33 months (case 1: 6 months, case 2: 17 months, case 3: 77 months). Long-term follow-up MRI revealed no adhesion of the spinal cord or recurrence of syringomyelia.

### Characteristics of syringomyelia before surgery

Syringomyelia was noted in all three cases rostral to the SAW (Fig. 2A, C). In case 1, the syrinx affected levels C6–T6 (length 14.5 cm, maximal AP diameter 6 mm, and maximal lateral diameter 5.18 mm) (Table 3). In case 2, levels C5–C7 was affected (length 3.4 cm, maximal AP diameter 3.4 mm, and maximal lateral diameter 4.7 mm) (Table 3). In case 3, levels C1–T8 was affected (length 26 cm, maximal AP diameter 5.5 mm, and maximal lateral diameter 4.4 mm) (Table 3).

**Table 3:** Characteristics of syringomyelia before and after surgery

Syrinx	Case 1	Case 2	Case 3
Affected levels	C6-T6	C5-C7	C1-T8
Length b.s. (cm)	14.5	3.4	26
Length a.s. (cm)	0	2.4	0
Maximal AP diameter b.s. (cm)	0.6	0.34	0.55
Maximal AP diameter a.s. (cm)	0	0.34	0
Maximal lateral diameter b.s. (cm)	0.53	0.47	0.44
Maximal lateral diameter a.s. (cm)	0	0.4	0
Volume b.s. (cm^3^)	1.425	0.348	2.378
Volume a.s. (cm^3^)	0	0.149	0

*b.s.* before surgery, *a.s* after surgery

The volume of the syrinx was 1.425 cm^3^, 0.348 cm^3^, and 2.378 cm^3^.

### Characteristics of syringomyelia after surgery

In two cases (case 1 and case 3), syringomyelia was not detected 3 months after surgery.

In case 2, remnant syringomyelia was detected 1 year after surgery but with decreased size and volume (length 2.4 cm, maximal AP diameter 3.4 mm, and maximal lateral diameter 4 mm) (Table 3). The volume also decreased from 0.348 to 0.149 cm^3^ (Table 3).

## Discussion

### Pathophysiology

The pia mater of the spinal cord is fixed to the dura mater on both sides by the dentate ligaments [23] and at the dorsal side by the septum posticum (or posterior septum) [21, 23, 25]. An intermediate leptomeningeal layer is closely attached to the inner aspect of the arachnoid [20].

The septum posticum is important to stabilize the dorsal spinal cord against the ventral displacement at the thoracic spine level of the normal thoracic kyphosis [7, 23], which could explain the predilection of an arachnoid web at the upper thoracic levels. Parkinson et al. [24] described the septum posticum as an “increasing number of overlying arachnoid membrane strands, which become thinner and fenestrated in lumbar level” [21, 24]. It had been theorized that this tissue, which is likely a mosaic of webs with “rogue arachnoid strands,” may, in cases of SAW, develop into a configuration that blocks the downward-moving flow of CSF.

Nisson et al. [21] pointed out that the underlying etiology of SAW remains unknown. Different authors hypothesized that SAW might be a subtype of arachnoid cyst [4, 14, 21, 23] or an arachnoid cyst that ruptured [13, 22], while other authors assumed remnant tissue from embryologic development [21].

According to a study [21], a change in flow dynamics may be a growth-signal activation stimulus, which triggers a process of arachnoid thickening and growth, leading to spinal cord compression and CSF flow obstruction.

Predisposing factors for SAW are a prior traumatic event or spine surgery, infection, and inflammation [23]. In our study, one patient had previous spine surgery due to cervical disk herniation. Zhang et al. [23, 33] concluded that 5 of 14 patients had a history of trauma or spine surgery, while Voglis et al. [31] reported a history of traumatic spine injury in 50%. Nisson et al. [21] reported a history of traumatic spine injury in 16% of cases and a history of previous surgery in 16% of cases.

Some authors [3, 14] mentioned that a change of arachnoid after infection or trauma might lead to SAW. In a histopathological analysis [6] of SAW connective tissue, small numbers of CD3+ T cells had been found. In our study, one patient had a post-Lyme disease status. In this patient, we sent tissue of the web for pathological analysis and learned that CD3+ T cells were found in small numbers (similar to Chang et al. [6]), which might support the hypothesis of an underlying inflammatory process resulting in consecutive fibrosis of connective tissue [6, 13].

Another finding is small ossifications of ligamentum flavum at the level corresponding to the location of SAW [6], which supports the hypothesis of potential infection from the epidural to the subarachnoid space [6, 14]. SAW seems to be a specific form of arachnopathy.

Also, a forceful CSF flow, which results in arachnoid herniation but not spinal cord herniation, is a characteristic of ISCH and has been discussed.

### CSF dynamic and syringomyelia in SAW

Greitz et al. [10] defined syringomyelia as a fluid-filled cavity originating in the central canal or spinal cord tissue and other authors [17] reported that several cases with idiopathic syringomyelia, arachnoid webs, pouches, and cysts may play an important role [17, 27].

This study analyzed the management of SAW in patients with syringomyelia. Voglis et al. [31] concluded in their multicenter study that 83% of patients with SAW had syringomyelia. In comparison, in the study of Nisson et al. [21], 67% of patients had a syrinx, and in the study of Laxpati et al. [16], only 17%.

In our study, the syrinx extended rostrally to the SAW in all three cases. Chang et al. [6] concluded that the location of the syrinx is not a reliable indicator of the site of arachnoid pathology. Klekamp [15] summarized that the syrinx extended rostrally to the lesion in 47% of cases, caudally in 24%, and both directions in 29%. Voglis et al. [31] concluded in their study that in patients with SAW and syringomyelia that in 33%, the syrinx was above the SAW. In 25%, it was at the same level, and in 25%, it was below the SAW. The location of the syringomyelia may be either rostral or caudal to the SAW because the specific structure of the arachnoid web can promote a one-way flow of CSF and unidirectional syringomyelia [23]. If the CSF pressure on the rostral side (P_R_) of the SAW is greater than on the caudal side (P_C_), the syrinx is formed caudal to the membrane [29]. If P_R_ is less than P_C_ (like in our cases), the syrinx is formed rostral to the SAW [29].

In our study, the syrinx involved between 2 and 14 levels before surgery. The length ranged from 3.4 to 14.5 cm, the maximal AP diameter ranged from 3.4 to 6 mm, whereas the maximal lateral diameter ranged from 4.4 to 5.2 mm. The volume of the syrinx varied before surgery from 0.348 to 2.378 cm^3^.

Heiss et al. [12] concluded in their study about the pathophysiology of primary spinal syringomyelia (syringomyelia associated with spinal pathology) that the mean length of the syrinx was 17.3 ± 12.2 cm and the mean diameter was 7.1 ± 2.8 mm.

We showed that the syrinx could no longer be detected in two cases after surgery. In one case, the length was reduced from 3.4 to 2.4 cm and the volume from 0.348 to 0.149 cm^3^.

In the Heiss et al. [12] study, the mean length of the syrinx after surgery was 11.2 ± 12.4 cm, and the mean diameter was 3.6 ± 3.8 mm.

### Pathophysiology of syringomyelia in SAW

Different authors presented several theories of the pathophysiology of syringomyelia in different diseases [10, 12, 15, 27]. In our study, 2.22% of patients (3/135) with syringomyelia who received surgery had SAW as the origin of syringomyelia. Regarding the syrinx in the case of SAW, we want to present the main hypothesis.

Chang et al. [6] hypothesized that an oblique arachnoid web with a small opening at one end of the oblique septum might create a one-way valve mechanism [6, 14] leading to the selective blockage of rostral movement. According to Heiss et al. [12, 27], the subarachnoid block increases the subarachnoid pulse pressure above the block, producing a pressure differential across the obstructed segment, which results in syrinx formation.

Greitz’s theory [10] of the “Venturi effect” (or intramedullary pulse pressure theory) regarding the development of syringomyelia is largely accepted [11]. It implies that SAW disturbs or interrupts the transmission of the systolic pulse pressure wave of cranial intramedullary CSF [1, 6, 10, 11, 19, 23]. This creates a pressure gradient between the intramedullary compartment of the spinal cord and the subarachnoid space, leading to the accumulation of extracellular fluid and cord dilation [10, 27].

Greitz et al. [10] concluded that since the Bernoulli theorem states that the total mechanical energy of flowing fluids remains constant, it implies that the increased fluid velocity at a narrowed flow channel decreases the pressure in the fluid.

Greitz et al. [10] also concluded that the jet of CSF flow causes a “suction effect” in the CSF spaces, with “centrifugally directed transmedullary pressure gradients that distend the cord at and immediately below the subarachnoid encroachment” [10]. These result in cavitation within the spinal cord [3].

By contrast, it has been hypothesized that the blockage of the fast components of CSF flow causes a pressure drop in the subarachnoid space distal to the blockage, thereby producing a pressure gradient between the inside and outside of the spinal cord, with the higher pressure being inside. The repetitive generation of this pressure gradient is the driving force of syrinx formation.

The Virchow-Robin space seems to play an important role as a pathway for CSF into the spinal cord [6, 8, 12].

### Symptoms

In our study, all patients had paresthesia or dysesthesia of the arm or the leg, while two patients presented with palsy (one of the arm and one of the leg), and one patient also had thoracic pain.

Nisson et al. [21] reported in a review of 19 articles that 67% of patients with SAW had weakness, 65% numbness and/or sensory loss, and 19% incontinence [13, 21].

In 81% of patients, symptoms predominated in the lower extremities, in 42% back/trunk, and 16% upper extremities [21]. On the day of surgery, half of the patients had symptoms for 1 year or longer [21].

Other authors reported additional symptoms such as progressive myelopathy [5, 23], back pain [11, 23], clonus [3, 11], hyperreflexia, and the Schiff-Sherrington phenomenon [11].

### Radiological diagnosis and radiological FU

Hamilton et al. [11] and Buttiens et al. [5] concluded that MRI is the gold standard for diagnosing SAW with the pathognomonic so-called “scalpel sign” (or scalpel blade sign) [21] because of the resemblance of the silhouette of a surgical scalpel blade [11]. However, it might be difficult for MRI to visualize focal arachnoid lesions [6].

In our study, all patients had the pathognomonic scalpel sign, with anterior displacement of and caliber variation (the mean caliber variation was 3 mm) of the spinal cord.

It might be challenging to differentiate between SAW and other diseases with a ventral displacement of the spinal cord.

The “scalpel sign” in the case of SAW can be differentiated from the “C-shape” in the case of ISCH and the “concave aspect of the posterior spinal cord” in the case of an arachnoid cyst [28]. The SAW and spinal cord herniation can be reliably distinguished on imaging by scrutinizing the nature of the dorsal indentation and the preserved ventral CSF flow [14, 23, 28]. In rare cases, an “upside-down scalpel sign” had been described [4].

An important sign to identify the location of SAW that causes syringomyelia is the termination of a syrinx cavity and a ventral displacement of the spinal cord next to the termination of the syringomyelia [1, 29].

In T2-weighted constructive interference in steady-state sequences or true FISP, it might be possible to visualize the web [4, 5, 23]. In our series, it was possible to visualize the SAW in T2 true FISP MRI. Chang et al. [6] concluded that localizing the location of CSF blockage precisely limits the extent of resection.

One crucial characteristic of SAW is preserved, but disturbed CSF flow [11, 32]. Ben Ali et al. [3] concluded that CINE (cardiac-gated phase contrast cine-mode MRI in multiple axial planes) could better identify and correctly localize the SAW and demonstrate that a one-way valve linked the flow of CSF because of the web.

Maurer et al. [18] and Hamilton et al. [11] mentioned using cardiac-gated phase contrast MRI (PC-MRI) of CSF flow in the spine. Chang et al. [6] could demonstrate a depression of caudorostral flow in the dorsal subarachnoid space at the level of SAW on PC-MRI. They demonstrated that the caudorostral CSF flow in the dorsal subarachnoid space was normalized after surgery.

One question of our study was the outcome of syringomyelia in SAW after surgery. In our study, we demonstrated that after the dissection of SAW, the syrinx was no longer detectable in two cases and had become smaller in one case.

Also, CT-myelography might be helpful for the diagnosis of SAW [14, 21, 23, 25].

### Treatment and outcome

Ben Ali et al. [3] concluded that the decision for therapy should be individualized, and the management of SAW ranges from conservative management (with regular imaging and neurophysiology) to surgical excision, where in the latter, the extent of excision remains the subject of debate. In cases of syringomyelia, myelomalacia, myelopathy, worsening of symptoms, and/or worsening of potentials in electrophysiology, surgery is necessary, analogous to other spinal cord pathologies.

In our series, two patients underwent laminoplasty (one patient at two levels and one at one level with undercutting of both adjacent laminae) and one laminectomy at one level.

Nission et al. [21] summarized in their review that in 89% of cases, laminectomy was performed, and in 12%, hemilaminectomy or MIS hemilaminectomy. Ben Ali et al. [3] concluded that intraoperative ultrasound may help to visualize the pathology.

We performed an excision of the arachnoid web in all cases. In contrast, in the literature, in 84%, an excision of the arachnoid web was performed, and in 5%, an additional myeloscopy [21]. In 10%, instead of excision, a shunt was placed, and in 7%, a stent was placed [21]. Aiyer et al. [1] pointed out that treatment options besides excision included duraplasty, and syringosubarachnoid [1, 9] and syringopleural shunts.

In the opinion of Park et al. [23], the extent of resection should be minimized to avoid secondary iatrogenic arachnoid adhesion. Vergara et al. [30] presented two cases of minimally invasive resection of SAW. Percutaneous intrathecal catheterization of the arachnoid web was reported in one case [23, 26].

Dissection of the arachnoid web usually shrinks the syrinx and improves symptoms [6]. It had been reported that, after surgery, 91% of patients [21] showed neurological improvement, 5% showed no change, and 5% displayed worsening [21]. However, the main goal of surgery is to prevent the progression of symptoms [14]. In our series, the symptoms of all three patients improved, but remnant symptoms remained.

## Conclusion

In summary, we demonstrated that 2,22% of cases of syringomyelia, which are not associated with neoplastic lesions, are associated with SAW. Surgery is a safe way to treat SAW. Even though syringomyelia usually improves on MRI and symptoms improve, remnant symptoms might remain. Dissection of the arachnoid membrane should be the first choice, with the goal of treating the origin of the disease (similar to the strategy used for other diseases leading to syringomyelia).

We want to advocate for clear criteria for the diagnosis of SAW and a standardized diagnostic (MRI including TrueFISP and CINE) in future studies.

The main limitation of this study is the small number of patients, due to the rare prevalence of SAW.

## Data Availability

All data generated or analyzed during the current study are included in this article.
